# Cockpit-Llama: Driver Intent Prediction in Intelligent Cockpit via Large Language Model

**DOI:** 10.3390/s25010064

**Published:** 2024-12-25

**Authors:** Yi Chen, Chengzhe Li, Qirui Yuan, Jinyu Li, Yuze Fan, Xiaojun Ge, Yun Li, Fei Gao, Rui Zhao

**Affiliations:** 1 College of Automotive Engineering, Jilin University, Changchun 130025, China; chenyi1522@mails.jlu.edu.cn (Y.C.); licz24@mails.jlu.edu.cn (C.L.); yuanqr23@mails.jlu.edu.cn (Q.Y.); lijy1522@mails.jlu.edu.cn (J.L.); fanyz23@mails.jlu.edu.cn (Y.F.); gexj23@mails.jlu.edu.cn (X.G.); 2 Graduate School of Information and Science Technology, The University of Tokyo, Tokyo 113-8654, Japan; li-yun@g.ecc.u-tokyo.ac.jp; 3 National Key Laboratory of Automotive Chassis Integration and Bionics, Jilin University, Changchun 130025, China

**Keywords:** intelligent cockpit, large language model, intent prediction, human–machine interaction

## Abstract

The cockpit is evolving from passive, reactive interaction toward proactive, cognitive interaction, making precise predictions of driver intent a key factor in enhancing proactive interaction experiences. This paper introduces Cockpit-Llama, a novel language model specifically designed for predicting driver behavior intent. Cockpit-Llama predicts driver intent based on the relationship between current driver actions, historical interactions, and the states of the driver and cockpit environment, thereby supporting further proactive interaction decisions. To improve the accuracy and rationality of Cockpit-Llama’s predictions, we construct a new multi-attribute cockpit dataset that includes extensive historical interactions and multi-attribute states, such as driver emotional states, driving activity scenarios, vehicle motion states, body states and external environment, to support the fine-tuning of Cockpit-Llama. During fine-tuning, we adopt the Low-Rank Adaptation (LoRA) method to efficiently optimize the parameters of the Llama3-8b-Instruct model, significantly reducing training costs. Extensive experiments on the multi-attribute cockpit dataset demonstrate that Cockpit-Llama’s prediction performance surpasses other advanced methods, achieving BLEU-4, ROUGE-1, ROUGE-2, and ROUGE-L scores of 71.32, 80.01, 76.89, and 81.42, respectively, with relative improvements of 92.34%, 183.61%, 95.54%, and 201.27% compared to ChatGPT-4. This significantly enhances the reasoning and interpretative capabilities of intelligent cockpits.

## 1. Introduction

Automotive intelligent cockpits are equipped with advanced hardware and software systems, providing human–machine interaction, network services, and scenario expansion capabilities that integrate humans, machines, and the environment. These cockpits offer a comprehensive experience for drivers and passengers, including safety, intelligence, efficiency, and comfort. Based on the type of functionality provided, the interactive functions of intelligent cockpits can be divided into three dimensions: driving control, riding comfort, and infotainment. The driving control domain focuses on improving intelligent vehicle driving quality, the riding comfort domain aims to enhance the passenger experience, and the infotainment domain provides value-added information services and entertainment. According to the level of intelligence, the evolution of intelligent cockpits from “traditional function realization” to “comprehensive intelligent cognition” is divided into five levels (L0 to L4): Functional Cockpit, Perception Intelligent Cockpit, Partially Cognitive Intelligent Cockpit, Highly Cognitive Intelligent Cockpit, and Fully Cognitive Intelligent Cockpit [1]. Among these, proactive interaction capabilities are the key to advancing cockpit intelligence. Its working mechanism generally involves: processing and analyzing data by the intelligent system, predicting the functions the user might need in the current scenario, determining the composition and importance of these functions, and then interacting with the user through appropriate means to provide services [2]. In this entire interaction process, intent prediction serves as the system’s core “brain”, and accurate prediction of user functional needs forms the basis of proactive cockpit interaction.

Human–cockpit interaction describes how humans interact with the cockpit, which can be divided into explicit and implicit interactions [3]. Explicit interaction involves users consciously and clearly expressing their intentions and needs, characterized by certainty and directness. Examples include voice-based interaction [4,5,6,7], haptic-based interaction [8,9,10,11], and gesture-based interaction [12,13,14], etc. Implicit interaction, on the other hand, focuses on non-direct user interactions, detecting and interpreting non-specific behaviors such as body movements, physiological signals, facial expressions, and environmental factors to intelligently understand user states and needs and respond autonomously [15]. Achieving higher-level intelligence relies on accurately understanding implicit interaction intentions. However, the aforementioned implicit intention inference methods face two limitations. First, traditional rule-based or data-driven lightweight model approaches lack scalability and comprehension capabilities, only applicable to independent, single-task intent prediction. In the cockpit environment, driving intentions, comfort, and information interactions are closely interwoven. Driver operations involve not only basic driving tasks but also controlling systems such as air conditioning, music, and navigation to meet comfort and information needs. Cockpits encompass dozens of different types of intentions, such as adjusting seat positions, changing air conditioning temperatures, making calls, playing music, or interacting with the navigation system during driving. These actions require coordination between multiple intelligent systems, making it difficult for single-task approaches to support high-level intelligence. Second, the reasoning and decision-making process of the entire system lacks transparency, resembling a “black box” operation, and lacks human social context knowledge, making it challenging to fully analyze human behavior and difficult for humans to understand.

The rapid development of large language models (LLMs) brings hope for addressing these challenges. LLMs possess strong generalization and common-sense reasoning abilities, allowing them to infer unseen information. Since they are pre-trained on extensive interdisciplinary data, including social interactions and human behavior, LLMs have prior common knowledge about the human world, potentially bridging the social intelligence gap in behavior prediction systems. LLMs have been used for predictive tasks in various fields, such as predicting future vehicle behaviors in autonomous driving, including lane changes, turns, and braking [2,16,17,18]. Compared to autonomous driving, cockpit intentions are more complex, encompassing three domains—driving control, riding comfort, and infotainment—with dozens or even nearly a hundred specific intentions. Moreover, most intention prediction works are still independent single tasks, while driver intentions are interrelated and holistic. Single-task predictions lead to inaccurate intention forecasts, a lack of coordination between multiple intentions, and potential conflicts.

This paper further proposes a comprehensive multi-task intention prediction method for intelligent cockpits based on LLMs, named Cockpit-Llama. Its prediction scope covers dozens of intentions in driving control, riding comfort, and infotainment. As LLMs are pre-trained on broad interdisciplinary data, including social interactions and human behavior, they have prior common knowledge about the human world. Utilizing this general knowledge, Cockpit-Llama achieves accurate multi-functional intention prediction within the cockpit. To ensure the rationality and accuracy of Cockpit-Llama’s predictions, we construct a dedicated multi-attribute cockpit dataset for cockpit intention prediction to fine-tune the model. During fine-tuning, adapter methods were used to optimize model parameters and reduce training costs. The main contributions of this work can be summarized as follows:

(1) **Dataset Construction**: A novel dataset is constructed containing approximately 60,000 sequences of multi-attribute states (including driver emotional states, driving activity scenes, vehicle motion states, body states, multimedia states and external environment) and driver historical interaction information, comprehensively reflecting the relationship between the driver’s future intentions, historical interaction sequences, and driving context.

(2) **Novel Prediction Model**: A novel prediction model named Cockpit-Llama is proposed, comprising three main components: modal data encoder, LLM backbone, and behavior intent decoder, specifically designed for the intention prediction task in intelligent cockpits. The model is fine-tuned on the constructed dataset. During fine-tuning, the LoRA adapter method is used to efficiently optimize model parameters, significantly reducing training costs. Cockpit-Llama demonstrates excellent performance in intent prediction across various scenarios (such as suburb drive, commute work, etc.), showcasing its strong generalization and reasoning capabilities.

(3) **Performance Evaluation**: Extensive experimental evaluation demonstrates that Cockpit-Llama outperforms large language models such as ChatGPT-4 and ChatGLM-3 in driver behavior prediction, achieving significant performance improvements. Compared to ChatGPT-4, BLEU-4 improved by approximately 92.34%, ROUGE-1 improved by 183.61%, ROUGE-2 by 95.54%, and ROUGE-L by 201.27%.

## 2. Related Works

### 2.1. Intelligent Cockpit Intent Prediction

Currently, intelligent cockpit intent prediction technology has been applied to varying degrees in driving control, riding comfort, and infotainment. In driving control, interaction research mainly focuses on predicting driver safety intentions derived from cognitive states. By monitoring driver facial expressions, eye movement, heart rate, and driving behavior changes, models can infer whether the driver is distracted or fatigued, allowing for timely safety interventions. Zhang et al. [19] used binary logistic regression and Fisher discriminant analysis to predict normal and distracted driving for safety warnings. Tran et al. [20] employed convolutional neural networks (CNN) to predict the driver’s distraction and fatigue states, effectively adapting to changes in lighting and shadows. Fasanmade et al. [21] developed a dynamic Bayesian (DDB) distraction severity classification model based on fuzzy logic, which predicts the severity of driver distraction by inputting the driver’s facial direction, activities, hand movements, and previous distraction history, enabling timely preventive measures. Riaz et al. [22] proposed a fuzzy logic-based driver distraction assessment system that calculates the driver’s level of distraction using fuzzy set algorithms. Additionally, they developed an emotion-enabled cognitive driving assistance model (EECDAM) to detect the driver’s emotional intensity and support the safety of driving decisions. Teyeb et al. [23] designed a multi-variable system based on fuzzy logic to infer driver vigilance levels through eye blinking analysis and head posture estimation, Xiao et al. [24] proposed a transfer learning-based method that utilizes driver facial images to recognize emotions in on-road environments.

Research in the riding comfort domain mainly focuses on emotion-driven proactive soothing interactions and intention prediction for vehicle component adjustments. In emotion-driven proactive soothing interactions, Li et al. [25], proposed using convolutional techniques to take driver facial expressions and cognitive process features as inputs for predicting driver emotions, thereby achieving affective human–machine interaction. Oehl et al. [26] proposed using self-assessment models to predict driver emotions based on the force exerted by the driver on the steering wheel. In vehicle component adjustment intention prediction, Hu et al. [27] proposed a dual-model coupling method, combining a habitual temperature prediction model and a time-series temperature prediction model, to predict air conditioning settings in real time.

In the infotainment domain, current research includes situational awareness, navigation intent prediction and music recommendation. Ferenc et al. [28] explored the effectiveness of fuzzy logic in context awareness prediction, using a set of 14 key prediction factors, including decision time, significance, eye-related indicators, and driver experience, to achieve accurate context awareness assessment. Li et al. [29] constructed a fuzzy inference system (FIS), using point-of-interest (POI) data from navigation maps as semantic cues to effectively infer various driving environments around the vehicle, such as shopping areas, tourist spots, public stations, service areas, and safety zones. Ye et al. [30] used power-law distribution (PD) to model user check-in behavior and proposed a collaborative point of interest (POI) algorithm based on geographical influence for prediction and recommendation using naive Bayes (NB). Sayak et al. [31] proposed a music recommendation method using content-based filtering and K-means clustering. However, these methods have inherent limitations. On one hand, the entire reasoning process resembles a “black box”, lacking intuitive interpretability and transparency. Additionally, they lack human social background knowledge, making it challenging to comprehensively analyze human behavior effectively, and difficult for humans to understand.

### 2.2. Large Language Models

Currently, LLMs have made rapid advancements, demonstrating significant potential in simulating human intelligence. These models are trained on vast amounts of internet data to understand and generate human-like text, showcasing outstanding performance in natural language processing. One of the most remarkable features of LLMs is their emerging capabilities, such as in-context learning (ICL), following instructions, and chain-of-thought (CoT) reasoning. Generative Pre-trained Transformers (GPT) [32] represents a pioneering effort, proposing the use of GPT to solve text understanding and generation tasks. Subsequent versions, GPT-3.5 and GPT-4 [33], have also demonstrated impressive conversational and reasoning abilities. Recently released LLMs, such as PaLM [34], Vicuna [35], LLaMA and LLaMA 2 [36,37], generate corresponding textual feedback based on human-followed instructions, enhancing the instruction-following capability of LLMs.

LLMs have been applied in multiple fields, including predicting the future behavior of autonomous vehicles, such as lane changes, turns, and braking. Zhao et al. [16] proposed an LLM model for autonomous driving decision-making, capable of predicting vehicle paths and performing motion planning accordingly. Xu et al. [17] introduced a multi-modal LLM-based end-to-end autonomous driving system, which can predict turning behaviors in an interpretable manner. Compared to autonomous driving, intent prediction in the cockpit is more complex, covering three domains: driving control, ride comfort, and infotainment, with dozens or even nearly a hundred specific intentions. In the cockpit, LLMs are currently mainly applied to single tasks, such as the HiVeGPT [38], which supports dialogue and facilitates interaction between human drivers and driving assistance systems. However, these intent predictions remain independent single tasks, whereas driver intent is interrelated and holistic. This may lead to inaccurate intent predictions, lack of coordination between multiple intents, and potential conflicts.

## 3. Methodology

**Figure 1:**
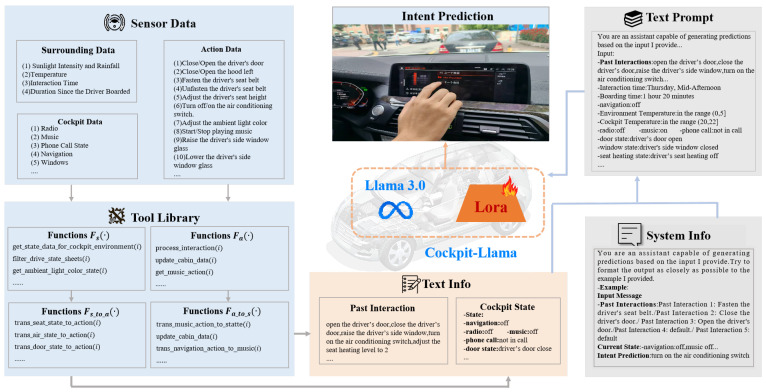
The overview of Cockpit-Llama.

This section proposes an LLM-based comprehensive multi-task intent prediction method for intelligent cockpits, Cockpit-Llama, which takes vehicle-centered human–vehicle interaction behaviors and multi-attribute states as inputs, and outputs the driver’s future intent. Figure 1 shows an overview of Cockpit-Llama, which mainly consists of three parts: multi-attribute cockpit dataset construction, Cockpit-Llama model architecture design and model fine-tuning strategy.

### 3.1. Multi-Attribute Cockpit Dataset Construction

Due to the scarcity of cockpit data, publicly available proprietary datasets for driver behavior prediction are almost nonexistent. Therefore, we specifically construct a novel multi-attribute cockpit dataset for driver behavior intent prediction. The participants include five laboratory members and fifteen recruited individuals. Data collection is conducted over the course of one month with five experimental vehicles, and each participant conducts at least one week of natural driving experiments. During the driving process, we collect a large amount of human–vehicle interaction data through embedded points in event tracking technology and vehicle status data via the Controller Area Network (CAN) bus. Additionally, after each trip, we infer the driver’s emotional state based on feedback from the driver and emotional records provided by the co-driver. By integrating and processing these multi-source data, we ultimately construct a large-scale novel cockpit dataset. This dataset includes human-cockpit historical interactions and multi-attribute states (including driver emotional states, driving activity scenarios, vehicle motion states, body states, multimedia states and external environment), which are used for fine-tuning Cockpit-Llama. In this study, we design a toolkit to pre-process, augment, pair the data, and package it into a text format for model training.

#### 3.1.1. Sensor Data Pre-Processing

Our goal is to process raw sensor data into the desired large-scale cockpit dataset. We deploy various sensors on five vehicles, including temperature sensors, humidity sensors, weather sensors, etc., to collect a large amount of raw sensor data. We also design a toolkit containing a set of functional functions to extract and integrate information about the cockpit, environment, and driver interactions from the raw sensor data, transforming them into model-learnable types. Specifically, we use the state extraction function $F_{s}\left(\cdot \right)$ from the toolkit to extract sensor data about the cockpit and the environment. By processing this data, we obtain information about the status of various in-cabin devices and the surrounding environment of the vehicle. Additionally, we use the driver behavior detection function $F_{a}\left(\cdot \right)$ from the toolkit to extract the corresponding driver interaction data.

#### 3.1.2. Data Augmentation

Due to limitations in technology and other conditions, the collected state data and behavior data often fail to comprehensively reflect the complexity of the driving environment. In other words, the existing behavioral attributes and state attributes are insufficient to fully characterize cockpit behaviors and their contexts. Therefore, in the research of cockpit data, the transformation between state and behavior is crucial to enhance data attributes and scale. In this work, we further processed the pre-processed data to perform data augmentation.

**State Attribute to Behavior Attribute Transformation.** The existing behavior attributes mainly reflect the driver’s usage of cockpit infotainment systems, such as radio, music, calls, navigation, and functional settings, lacking the capture of behaviors like air conditioning adjustment, door operations, window adjustment, and seat adjustment. To capture these behaviors, we construct a state-to-behavior attribute transformation algorithm, $F_{s_{-}{\mathrm{to}}_{-}a}\left(\cdot \right)$. The specific process of transforming seat status into behavior is detailed in Algorithm 1. First, the algorithm initializes the sensor data $\delta_{tobs}$ at the current time $t_{obs}$ and the sensor data $\delta_{t_{obs-1}}$ at the previous time $t_{obs-1}$ (line 1). Then it traverses all state data and uses the state-to-behavior function $F_{s_{-}{\mathrm{to}}_{-}a}\left(\cdot \right)$ from the toolkit to extract the corresponding seat heating states $s_{bod,t_{obs}}^{seat}$ and $s_{bod,t_{obs}-1}^{seat}$ from the sensor data $\delta_{t_{obs}}$ at present time $t_{obs}$ and the sensor data $\delta_{t_{obs-1}}$ at previous time $t_{obs-1}$ (lines 2 to 3). The value of $s_{bod,t_{obs}}^{seat}$ can be 0, 1, 2, or 3, representing seat heating off, level one, level two, and level three heating, respectively. Then, further judge $s_{bod,t_{obs}}^{seat}$ and $s_{bod,t_{obs}-1}^{seat}$, when $s_{bod,t_{obs}}^{seat}$ and $s_{bod,t_{obs}-1}^{seat}$ are different, return the corresponding behavior value $h_{seat,t_{obs}}$ (lines 4 to 20). By analyzing the transformation of various state information within the cockpit through this algorithm, driver behaviors can be inferred, enriching the behavior data.
**Behavior Attribute to State Attribute Transformation.** Similar to the transformation from state attributes to behavior attributes, the existing state attributes are mainly limited to vehicle driving states and vehicle body states, lacking descriptions of the in-cabin device states, which can be inferred from behavior attributes. For instance, behaviors such as starting music playback indicate that the music state is on, while pausing music playback or ending the driving journey represents the music state being off. Therefore, when capturing the driver’s behavior of starting music playback, it can be transformed into the action of turning on the music player. Similarly, when capturing the driver’s behavior of stopping music playback, other common state attributes that can be inferred include radio, phone, and navigation driving scenes. The specific construction method is detailed in Algorithm 2. First, initialize the detected driver behavior ***H*** (line 1), then traverse all the behavior data ***H*** to extract multimedia-related behaviors (lines 2 to 3). Next, judge the corresponding value of $h_{mul,t_{obs}}$ and return the state $s_{mul,t_{obs}}$ corresponding to $h_{mul,t_{obs}}$ (lines 4 to 16). Algorithm 2 can convert cockpit behavior attributes into state attributes. By analyzing driver behavior data, the usage status of these devices can be inferred, enriching the state attributes.

**Algorithm 1** Seat heating state to action algorithm**Input:** present sensor data $\delta_{t_{obs}}$, previous sensor data $\delta_{t_{obs-1}}$
**Output:** The Action $h_{t_{obs}+1}$
1:Initialize $\delta_{t_{obs}}\neq \emptyset$,$\delta_{t_{obs-1}}\neq \emptyset$2:**for** t←0 in *N* **do**3:    $\delta \;\mathrm{Extract}\;s_{bod,t_{obs}}^{seat}\in \delta_{t_{obs}},s_{bod,t_{obs}-1}^{seat}\in \delta_{t_{obs-1}}$4:    **if** $s_{bod,t_{obs}}^{seat}=0$ **then**5:        **if** $s_{bod,t_{obs}-1}^{seat}\neq 0$ **then**6:           $h_{bod,t_{obs}}:=“Turn\;off\;the\;seat\;heating$”7:        **end if**8:    **else if** $s_{bod,tobs}^{seat}=1$ **then**9:        **if** $s_{bod,t_{obs}-1}^{seat}\neq 1$ **then**10:           $h_{bod,t_{obs}}:=“Adjust\;seat\;heating\;level\;to\;1$”11:        **end if**12:    **else if** $s_{bod,tobs}^{seat}=2$ **then**13:        **if** $s_{bod,t_{obs}-1}^{seat}\neq 2$ **then**14:           $h_{bod,t_{obs}}:=“Adjust\;seat\;heating\;level\;to\;2$”15:        **end if**16:    **else if** $s_{bod,tobs}^{seat}=3$ **then**17:        **if** $s_{bod,t_{obs}-1}^{seat}\neq 3$ **then**18:           $h_{bod,t_{obs}}:=“Adjust\;seat\;heating\;level\;to\;3$”19:        **end if**20:    **end if**21:**end for**


**Algorithm 2** Action to state algorithm**Input:** Action data ***H***
**Output:** State $s_{mul,t_{obs}}$
1:Initialize H≠∅2:**for** t←0 in *N* **do**3:    $\Delta \;\mathrm{Extract}\;h_{mul,t_{obs}}\in H$4:    **if** $h_{mul,t_{obs}}=“Play\;the\;music"\;\mathrm{or}\;“Continue\;the\;music"$ **then**5:        $s_{mul,t_{obs}}:=“Music\;On$”6:    **else if** $h_{mul,t_{obs}}=“Stop\;the\;music"$ **then**7:        $s_{mul,t_{obs}}:{=}^{n}Music\;Off"$8:    **else if** $h_{mul,t_{obs}=}“Play\;the\;radio$” **then**9:        $s_{mul,t_{obs}}:=“Radio\;On$”10:    **else if** $h_{mul,t_{obs}}=“Stop\;the\;radio$” **then**11:        $s_{mul,t_{obs}}:=“Radio\;Off$”12:    **else if** $h_{mul,t_{obs}}=“Enter\;the\;Navigation$” **then**13:        $s_{mul,t_{obs}}:=“Navigation\;On$”14:    **else**15:        $s_{mul,t_{obs}}:=“Navigation\;Off$”16:    **end if**17:**end for**


#### 3.1.3. Dataset Text Generation

In this work, the pre-processed data and augmented data are integrated to form a global feature set $X_{T}$, which consists of the multi-attribute states set $s_{tobs}$ and historical behavior sequences $h_{\left(t_{obs}-j\right):t_{obs}}$ of the driver at time *j*:

$$X_{T}=\left\{s_{t_{obs},}h_{\left(t_{obs}-j\right):t_{obs}}\right\}$$

where behavior sequence $h_{\left(t_{\mathrm{obs}}-j\right):t_{\mathrm{obs}}}=\left\{h_{t_{\mathrm{obs}}-j},h_{t_{obs}-j+1},\ldots ,h_{t_{obs}}\right\}$ records the specific contents of driver’s current interaction and the previous j historical interactions. Multi-attribute states set $s_{t_{\mathrm{obs}}}$ includes the driver emotional states $s_{\mathrm{emo}}$, driving activity scenarios $s_{\mathrm{sce}}$, vehicle motion states $s_{\mathrm{dri}}$, body states $s_{\mathrm{bod}}$, multimedia states $s_{\mathrm{mul}}$ and external environment $s_{\mathrm{env}}$:

$$s_{t_{\mathrm{obs}}}=\left\{s_{\mathrm{emo}},s_{\mathrm{sce}},s_{\mathrm{dri}},s_{\mathrm{bod}},s_{\mathrm{mul}},s_{\mathrm{env}}\right\}$$

Driver emotional states $s_{\mathrm{emo}}$ include seven types: anger, disgust, happiness, fear, sadness, surprise, and neutral:

$$s_{emo}=\left\{s_{\mathrm{emo}}^{\mathrm{anger}},s_{\mathrm{emo}}^{\mathrm{disgust}},s_{\mathrm{emo}}^{\mathrm{happiness}},s_{\mathrm{emo}}^{\mathrm{fear}},s_{\mathrm{emo}}^{\mathrm{sadness}},s_{\mathrm{emo}}^{\mathrm{surprise}},s_{\mathrm{emo}}^{\mathrm{neutral}}\right\}$$

Driving activity scenarios $s_{\mathrm{sce}}$ sre divided into six types: daily scenario, weekend travel scenario, far driving scenario, suburb driving scenario, commute work scenario, and commute home scenario.

$$s_{sce}=\left\{s_{\mathrm{sce}}^{\mathrm{daily}},s_{\mathrm{sce}}^{\mathrm{week}},s_{\mathrm{sce}}^{\mathrm{suberb}},s_{\mathrm{sce}}^{\mathrm{far}},s_{\mathrm{sce}}^{\mathrm{work}},s_{\mathrm{sce}}^{\mathrm{home}}\right\}$$

Vehicle motion states $s_{\mathrm{dri}}$ include vehicle speed, steering angle, braking, etc:

$$s_{\mathrm{dri}}=\left\{s_{\mathrm{dri}}^{\mathrm{speed}},s_{\mathrm{dri}}^{\mathrm{yaw}},s_{\mathrm{dri}}^{\mathrm{brake}}\ldots \right\}$$

Vehicle body states $s_{\mathrm{bod}}$ include air conditioning status, window openness, sunroof openness, door status, ambient light status, seat status, cabin temperature, etc:

$$s_{bod}=\left\{s_{\mathrm{bod}}^{\mathrm{air}},s_{\mathrm{bod}}^{\mathrm{window}},s_{\mathrm{bod}}^{\mathrm{sunroof}},s_{\mathrm{bod}}^{\mathrm{door}},s_{\mathrm{bod}}^{\mathrm{light}},s_{\mathrm{bod}}^{\mathrm{seat}},s_{\mathrm{bod}}^{\mathrm{temp}}\ldots \right\}$$

where air conditioning status values $s_{\mathrm{bod}}^{\mathrm{air}}$ include air conditioning mode, fan speed, and temperature. Seat status values $s_{\mathrm{bod}}^{\mathrm{seat}}$ include seat adjustment angle and seat heating level.

Multimedia states $s_{\mathrm{mul}}$ include music playback status, radio playback status, and navigation status:

$$s_{\mathrm{mul}}=\left\{s_{\mathrm{mul}}^{\mathrm{music}},s_{\mathrm{mul}}^{\mathrm{radio}},s_{\mathrm{mul}}^{\mathrm{navigation}}\right\}$$

External environment $s_{\mathrm{env}}$ includes external temperature, ambient light, and rainfall status:

$$s_{\mathrm{env}}=\left\{s_{\mathrm{env}}^{\mathrm{temp}},s_{\mathrm{env}}^{s\mathrm{unlight}},s_{\mathrm{env}}^{\mathrm{rainfall}}\right\}$$

**Figure 2:**
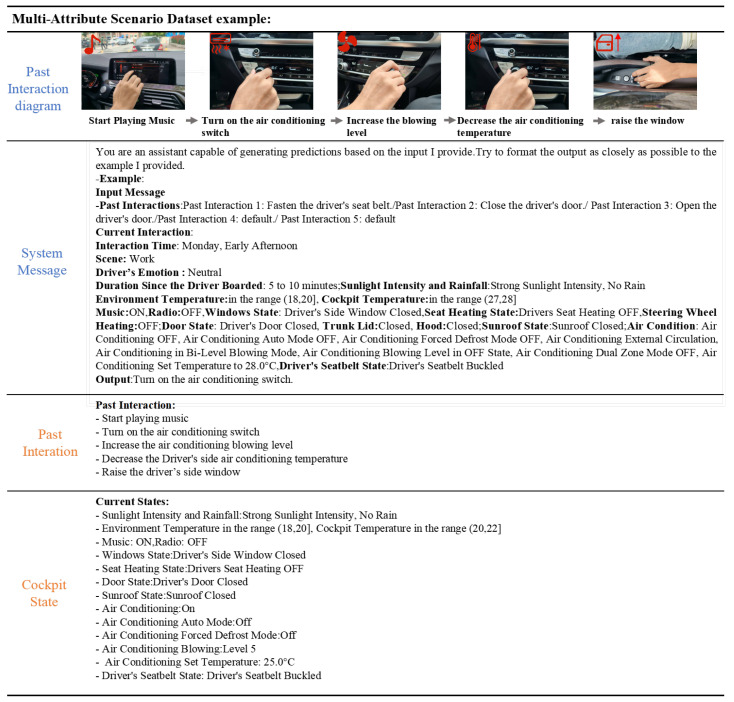
Example of multi-attribute cockpit dataset.

**Figure 3:**
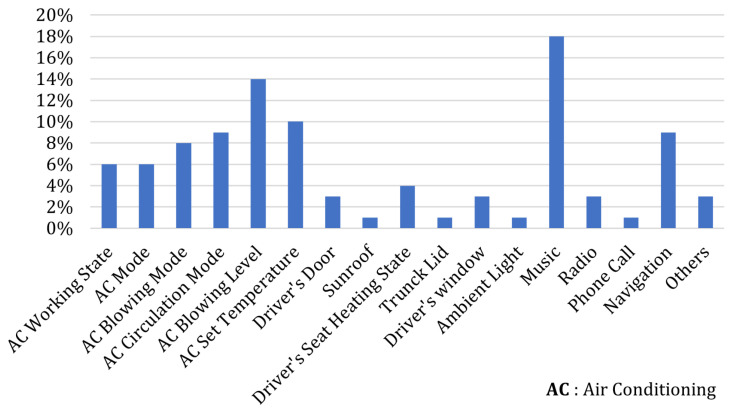
Statistical analysis of dataset intents.

To enable the model to produce higher-quality answers to prediction tasks, we also carefully design text instructions $X_{I}$ constitutes the input for constructing the cockpit instruction-following dataset. Finally, we combine the textual instructions $X_{I}$ and the global dataset $X_{T}$ content to form the model’s input dataset, which contains approximately 60k entries. As shown in Figure 2, we present an example sample from a multi-attribute scenario dataset. Moreover, we provide statistics on some representative behaviors from the dataset, as shown in Figure 3.

### 3.2. Cockpit-Llama Model Architecture Design

**Figure 4:**
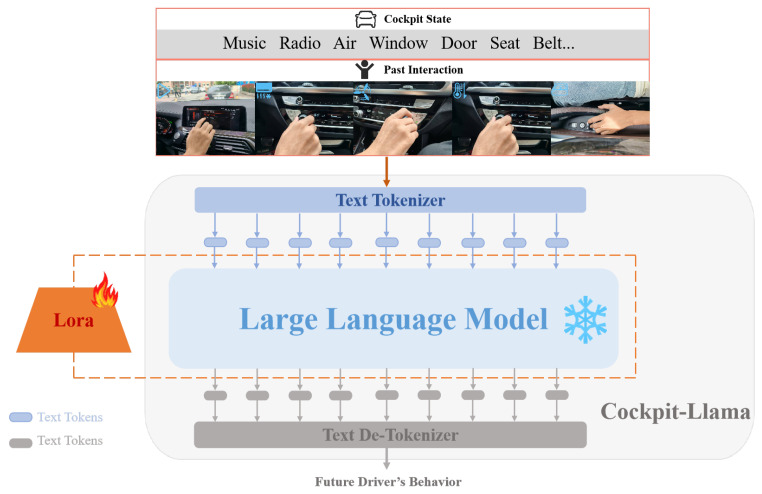
The overall architecture of Cockpit-Llama is divided into three parts: modal text data encoding, lightweight fine-tuning of LLM backbone using adapter, and driver behavior intent decoding.

As shown in Figure 4, the Cockpit-Llama model architecture mainly consists of three major components: modal text data encoding, lightweight fine-tuning for the LLM backbone, and driver behavior intent decoding. First, a text tokenizer is used to convert the text instructions into a format that the LLM can process, which is then input into the LLM for processing. Next, to ensure that Cockpit-Llama can precisely adapt to the current task, an adapter method is used to fine-tune the model, with adapters added to the LLM backbone. This adapter fine-tuning method enhances the LLM’s ability to understand and process text data, enabling it to generate effective prediction labels. It also optimizes the parameter size of the LLM, significantly reducing training costs while maintaining high performance and efficiency. Finally, the prediction labels generated by the LLM are decoded using a text detokenizer. To improve the efficiency of extracting inference results, driver behavior intents are decoded hierarchically, and during the decoding process, the predicted driver behavior intents are output in text format.

#### 3.2.1. Modal Text Data Encoding

The input to Cockpit-Llama consists of the global feature set $X_{T}$ and a piece of instruction text $X_{l}$ used to guide the large language model. The text-tokenizer $F_{\mathrm{token}}\left(\cdot \right)$ is used to convert all the contents of our constructed multi-modal dataset into corresponding text token $R_{L}$:

(1) $$R_{L}=F_{token}\left(X_{T}⊕X_{I}\right)$$

where $R_{L}$ belongs to the text embedding space, which can be understood and processed by the LLM backbone.

#### 3.2.2. Lightweight Fine-Tuning of LLM Backbone Using Adapters

**Figure 5:**
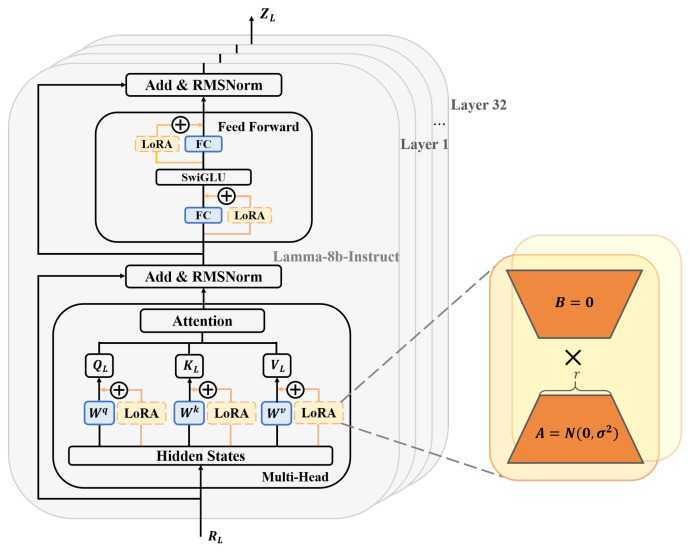
Lightweight fine-tuning with adapter for LLM backbone.

In this work, we use LoRA as an adapter to perform lightweight fine-tuning of the LLM backbone as shown in Figure 5. The core idea of the LoRA fine-tuning method is to dynamically adjust the model weights by adding low-rank matrices to the pre-trained model’s weight matrices. This fine-tuning method allows effective adjustment of the model’s performance for specific tasks using only a small number of additional parameters, while keeping the pre-trained model’s weight parameters unchanged.

We use Llama3-Instruct-8b as the LLM backbone, which consists of 32 transformer layers and has excellent semantic understanding and logical reasoning capabilities. In this work, LoRA is added to each linear layer of Llama3-8B-Instruct, including the multi-head attention (MHA) layer and feed-forward network (FFN) layer. Input tokens $R_{L}$ first enter the MHA layer. In this layer, LoRA is added to each attention matrix, which includes the query matrix $Q_{L}$,key matrix $K_{L}$,value matrix $V_{L}$.LoRA is a bypass module containing a down-projection matrix ***A*** and an up-projection matrix ***B***. During fine-tuning, both the input and output dimensions of the MHA layer remain unchanged, and the linear transformation matrices of each attention matrix are frozen. Only the additionally introduced down-projection matrix ***A*** and up-projection matrix ***B*** are trained, where ***A*** is initialized using a random Gaussian distribution and ***B*** is initialized with a zero matrix. The entire MHA mechanism computation process is as follows:

(2) $$\mathrm{MultiHead}\left(Q_{L,}K_{L,}V_{L}\right)=\mathrm{Concat}\left({\mathrm{head}}_{1,}......,{\mathrm{head}}_{h}\right)W^{0}$$

(3) $${\mathrm{head}}_{h}=\mathrm{Attn}\left(Q_{L,}K_{L,}V_{L}\right)=\mathrm{softmax}\left(\frac{Q_{L}K_{L}^{T}}{\sqrt{d_{k}}}\right)$$

(4) $$where\;\left\{\begin{array}{c} Q_{L}=\left(W_{h}^{q}+B_{h}^{q}A_{h}^{q}\right)Y_{L}\\ K_{L}=\left(W_{h}^{k}+B_{h}^{k}A_{h}^{k}\right)Y_{L}\\ V_{L}=\left(W_{h}^{v}+B_{h}^{v}A_{h}^{v}\right)Y_{L} \end{array}\right.$$

where $W_{h}^{q}\in R^{\left(d_{m}d_{k}\right)},W_{h}^{k}\in R^{\left(d_{m}d_{k}\right)},W_{h}^{\nu }\in R^{\left(d_{m}d_{k}\right)}$ are the linear transformation matrices for each attention head $Q_{L}$, $K_{L}$, $V_{L}$. $A_{h}^{q}\in R^{\left(d_{\mathrm{llama}},r\right)}$, $A_{h}^{k}\in R^{\left(d_{\mathrm{llama}},r\right)}$ are the corresponding down-projection matrices. $B_{h}^{q}\in R^{\left(r,d_{k}\right)}$, $B_{h}^{k}\in R^{\left(r,d_{k}\right)}$, $B_{h}^{v}\in R^{\left(r,d_{k}\right)}$ are the corresponding up-projection matrices. $W^{0}$ represents the transformed output matrix. Then, after a residual connection and layer normalization, we obtain $E_{L}$:

(5) $$E_{L}=\mathrm{RMSNorm}\left(H_{L}+\mathrm{MultiHead}\left(Q_{L,}K_{L,}V_{L}\right)\right)$$

Next, $E_{L}$ passes through the feed-forward network layer rFNN(·), which includes two fully connected layers and an activation function SwiGLU(·). LoRA is added to both fully connected layers, using the same training method as above: freezing the weight matrices of the two fully connected layers and training only the down-projection matrix ***A*** and up-projection matrix ***B***. After another residual connection and layer normalization, we obtain the output of this transformation layer ${\tilde{H}}_{L}$:

(6) $${\tilde{H}}_{L}=\mathrm{RMSNorm}\left(E_{L}+\mathrm{rFFN}\left(E_{L}\right)\right)$$

where ${\tilde{H}}_{L}$ serves as input tokens for next transformer layer and above operations are repeated. Finally, after passing through L=32 layers of transformers with added LoRA, predicted tokens $Z_{L}$ of LLM backbone are obtained:

(7) $$Z_{L}=\sum_{L=1}^{32}{\left(Transfomer\left({\tilde{H}}_{L}\right)\right)}^{L}$$

where $Z_{L}$ contains all reasoning information for the current task.

#### 3.2.3. Driver Behavior Intent Decoding

After obtaining the predicted token $Z_{L}$ generated by the LLM backbone, Llama3’s default detokenizer $f_{\mu }\left(\cdot \right)$ is used to decode $Z_{L}$ into human-readable language. This decoding method improves the transparency of Cockpit-Llama throughout the entire prediction process while ensuring the accuracy of the prediction results. The entire decoding process is represented as follows:

(8) $$h_{t_{obs}+1}=f_{\mu }\left(Z_{L}\right)$$

where, $h_{t_{obs}+1}$ represents the driver’s interaction behavior in the next frame, and is output in natural language form.

### 3.3. Model Fine-Tuning Strategy

Cockpit-Llama is fine-tuned using the instruction dataset constructed in this work to achieve driver behavior prediction. Considering that Cockpit-Llama’s output is in natural language, cross-entropy loss L(θ) is used to supervise the model’s inference output. The cross-entropy loss is defined as follows:

(9) $$L\left(\theta \right)=\sum_{i}^{N}p_{\theta }\left(h_{t_{obs+l}}\right)log\left(p_{\theta }\left(h_{t_{obs+l}}|X_{T}⊕X_{I}\right)\right)$$

**Figure 6:**
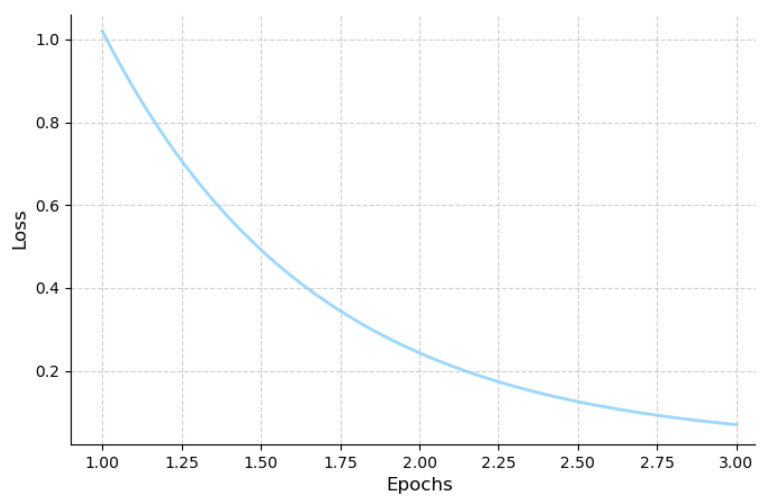
The loss curve variation during Cockpit-Llama training.

By minimizing this cross-entropy loss function, we can find the optimal parameters, allowing the model to better interpret or fit the data. As shown in the Figure 6, the cross-entropy curve during Cockpit-Llama fine-tuning converges around a loss value of 0.02.

During fine-tuning, to significantly reduce training costs, the LoRA method was used to effectively optimize the model parameter size while keeping the weights of the image tokenizer and the LLM backbone fixed. By minimizing cross-entropy loss, the LoRA weights were updated, enabling Cockpit-Llama to accurately infer future driver behavior.

## 4. Experiment

This section evaluates the performance of Cockpit-Llama through extensive experiments on the multi-attribute cockpit dataset. First, the experimental setup and evaluation metrics are introduced. Next, this method is compared with other baseline methods, demonstrating its superior performance in intent prediction. This work also designs a comparative experiment involving in-context learning and freeze fine-tuning to highlight the necessity of model fine-tuning and the efficiency of LoRA fine-tuning.

### 4.1. Experimental Setup

#### 4.1.1. Implementation Details

We split the entire cockpit dataset into a 4:1 ratio for the training and testing sets, fine-tuning the model on the training set and evaluating its performance on the testing set. To ensure a fair comparison with baseline methods, the models compared against Cockpit-Llama in the experiments were evaluated using the same test set. The initial learning rate was set to 5 ×$10^{-5}$ and gradually decayed to zero using cosine annealing. Considering the large number of parameters in the model, the LoRA fine-tuning method was used to optimize parameter size and reduce training costs. The rank r of the down-projection matrix was set to 8, targeting all linear layers in the LLM backbone of Cockpit-Llama. We performed 3 epochs of fine-tuning on four L20 (48 GB) GPUs, with a batch size of 2 for each GPU. The entire fine-tuning process was conducted on these GPUs.

#### 4.1.2. Evaluation Metrics

To quantitatively evaluate Cockpit-Llama’s performance in intent prediction, different evaluation metrics are used. For the intent prediction task, this study uses BLEU-4, Rouge-N, and Rouge-L as evaluation metrics. These are commonly used automatic evaluation metrics for assessing the similarity and quality of generated text in generative tasks (such as machine translation, text summarization, etc.) compared to reference texts. The specific roles and principles of these metrics are as follows:

1. BLEU-4: BLEU-4 [39] is a common metric for evaluating machine translation quality, measuring the similarity between source and target sentences. Its formula is:

(10) $$BLEU-4=BP\cdot exp(\sum_{i}^{N_{1}}w_{4}logP_{4})$$

where BP represents the brevity penalty, $w_{4}$ represents the weight proportion of the current word n-gram and $P_{4}$ represents the 4-gram precision.

2. ROUGE-N: ROUGE-N [40] measures the overlap of words between the model output and reference text, with higher scores indicating greater similarity:

(11) $$Rouge-N=\frac{Count_{match}\left(gram_{n}\right)}{Count_{output}\left(gram_{n}\right)}$$

where $Count_{match}\left(.\right)$ is the number of n-grams in Cockpit-Llama’s output that match the test set, and $Count_{output}\left(.\right)$ is the number of n-grams in the model’s output. In this experiment, we use ROUGE-1 and ROUGE-2 to evaluate performance.

3. ROUGE-L:

(12) $$ROUGE-L=\frac{\left(1+\lambda^{2}\right)I_{lcs}K_{lcs}}{I_{lcs}+\lambda^{2}K_{lcs}}$$

where $I_{lcs}$ represents the longest common subsequence match count, $K_{lcs}$ represents the longest common subsequence length proportion, and $\lambda^{2}$ is the adjustment factor.

### 4.2. Comparison with Baseline Methods

To demonstrate the superior performance of Cockpit-Llama in intent prediction, we compared it with the advanced baseline methods ChatGPT-4 and ChatGLM-3, which have shown outstanding performance in text understanding and logical reasoning. After fine-tuning with the constructed dataset, Cockpit-Llama significantly outperformed ChatGPT-4 and ChatGLM-3 on all metrics for the specific task of predicting driver behavior. This experiment demonstrates the superior performance of Cockpit-Llama. As shown in Table 1, Cockpit-Llama’s BLEU-4 score improved by 92.34% over ChatGPT-4 and 135.14% over ChatGLM-3. This score indicates a high similarity between Cockpit-Llama’s predicted driver behavior and the human-annotated driver behavior, far surpassing ChatGPT-4 and ChatGLM-3, demonstrating its accuracy and effectiveness in predicting driver behavior.

In the ROUGE-1 evaluation, Cockpit-Llama outperformed ChatGPT-4 by 183.61% and ChatGLM-3 by 211.41%. For ROUGE-2, Cockpit-Llama’s score was 95.54% higher than ChatGPT-4’s and 459.80% higher than ChatGLM-3’s. The ROUGE-n metric counts the number of overlapping units between the model’s predicted output text and the human-annotated output text, as well as the proportion of overlapping units in the human-annotated text. According to the experimental data, Cockpit-Llama achieved significantly higher recall rates compared to ChatGPT-4 and ChatGLM-3.

In terms of ROUGE-L, Cockpit-Llama outperformed ChatGPT-4 by 201.27% and ChatGLM-3 by 196.41%, indicating a high similarity between the longest common subsequences of the model’s predicted output text and the human-annotated text, significantly surpassing the two comparison models. Based on these four evaluation metrics, it is evident that Cockpit-Llama surpasses the other two models in both token repetition and longest common subsequence length, demonstrating its accuracy and reliability in driver behavior prediction tasks.

In summary, compared to the other two models, our Cockpit-Llama achieves superior results in driver intent prediction tasks. We attribute this to two main factors: On the one hand, Cockpit-Llama has 8 billion parameters and has been pre-trained on large-scale internet data, which provides it with prior knowledge about the world, enabling it to better understand and process language tasks. On the other hand, we fine-tune the model using a multi-attribute cockpit dataset specifically designed for driver intent prediction, which provides various contextual information, including driver historical interactions, driver emotional states, vehicle states, and external environments, to assist the model in making more accurate predictions.

### 4.3. In-Context Learning and Freeze vs. Cockpit-Llama (LoRA)

To further verify the compatibility and optimized design of our method, this experiment compared the lightweight fine-tuning method used in our model (LoRA) with Freeze fine-tuning and in-context learning.

As shown in Table 2, compared to the in-context learning method, Cockpit-Llama achieved an 80.36% higher BLEU-4 score, 120.75% higher ROUGE-1 score, 74.67% higher ROUGE-2 score, and 92.52% higher ROUGE-L score. Compared to Freeze fine-tuning, Cockpit-Llama achieved a 52.13% higher BLEU-4 score, 61.79% higher ROUGE-1 score, 41.54% higher ROUGE-2 score, and 60.27% higher ROUGE-L score.

It can be seen that using the LoRA fine-tuning method is more effective than using in-context learning or Freeze fine-tuning. Typically, Freeze fine-tuning only fine-tunes the fully connected layer parameters in the last few layers of the transformer while freezing all other parameters, which may lead to insufficient training and forgetting previously acquired capabilities. In-context learning, on the other hand, simply provides the model with a few examples, allowing the model to learn from these examples in context before making predictions, without changing the model parameters. This results in poor performance when dealing with complex scenarios like the intelligent cockpit environment.

The LoRA method, however, is a bypass adapter approach that decomposes the pre-trained model’s weights into low-rank matrices and adjusts the parameters of these matrices. This method reduces the training load while retaining training parameters, allowing the model to effectively adapt to complex scenarios.

### 4.4. Dataset Analysis and Visualization

**Figure 7:**
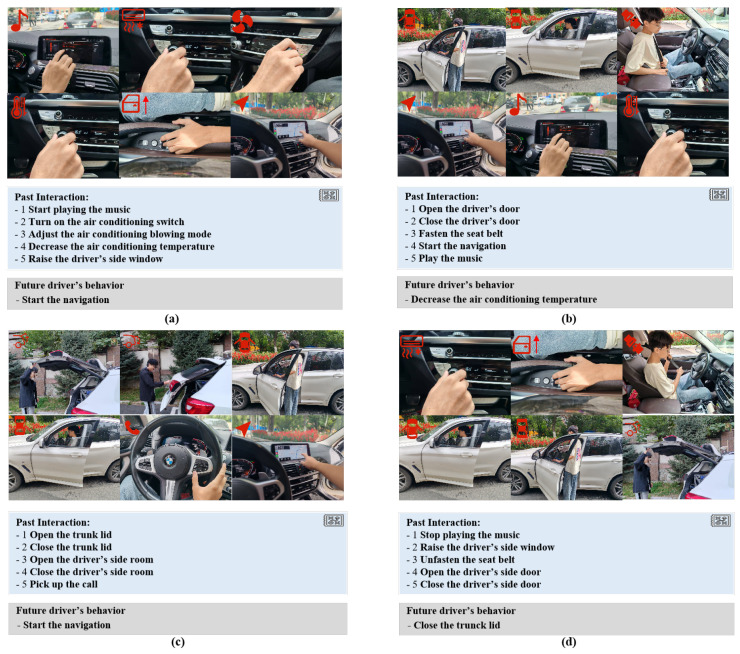
Visualization examples of Cockpit-Llama’s prediction process and interpretative language captions: (**a**) commute work scenario, (**b**) daily scenario, (**c**) far driving scenario, (**d**) weekend travel scenario.

To demonstrate the accuracy of Cockpit-Llama across multiple scenarios, Figure 7 presents four typical scenarios: commute work scenario, daily scenario, far driving scenario, and travel scenario. The figure visualizes the driver’s historical interactions and the model’s predicted future behavior. In the commute work scenario (Figure 7a), Cockpit-Llama accurately predicts that the driver will initiate navigation based on five interactions. In the daily scenario (Figure 7b), Cockpit-Llama predicts that the driver will likely lower the air conditioning temperature, also based on five interactions. Similarly, Figure 7c shows the far driving scenario where Cockpit-Llama predicts the driver will start the navigation. Finally, in the weekend travel scenario (Figure 7d), Cockpit-Llama predicts that the driver’s next action will be to open the trunk lid.

In summary, through quantitative analysis of Cockpit-Llama’s results in multiple experiments, its superior prediction performance has been demonstrated; through prediction examples and qualitative analysis in various typical scenarios, its superior generalization ability has been further demonstrated.

## 5. Conclusions

In this study, we propose a large language model specifically designed for predicting driver behavior, named Cockpit-Llama. This model is applied in a smart cockpit environment, integrating contextual information and historical interactions to provide accurate predictions of future driver intentions. To support the fine-tuning of Cockpit-Llama, we also construct a novel multi-attribute cockpit dataset, which includes extensive historical driver interactions and multi-attribute states. During the fine-tuning process, we adopt the LoRA method to optimize the model parameters and reduce training costs. Extensive experimental results demonstrate that Cockpit-Llama excels in predicting driver intentions, outperforming baseline models. Therefore, the LLM-based intelligent cockpit intent prediction method proposed in this work drives the transformation of human–cockpit interaction from isolated single-task to collaborative multi-task, from explicit passive to implicit proactive, promoting the cockpit’s evolution towards higher-level intelligence and significantly enhancing the user experience. Moreover, this method can also help drivers better respond to potential hazardous situations, improving driving safety.

**Limitations and Future Work:** Due to the limited size of the dataset, only about 60,000 samples were used to fine-tune Cockpit-Llama, and the inference time is relatively long due to the constraints of parameter size, making it challenging to meet the real-time requirements of commercial driving applications. Additionally, the current model may not fully capture the complexity of driver behavior and the various influencing factors. Future work should focus on constructing larger-scale datasets aiming to provide prior knowledge for the model. It should also explore using large language models to guide smaller ones to optimize inference time. Furthermore, integrating real-time data and additional contextual variables could further enhance the model’s predictive accuracy.

## Figures and Tables

**Table 1:** Baseline methods vs. Cockpit-Llama.

Method	Type	Evaluation			
		Bleu-4	Rouge-1	Rouge-2	Rouge-L
ChatGPT-4 [33]	LLM	37.08	28.21	39.32	27.03
ChatGLM-3 [41]	LLM	30.33	25.69	13.74	27.47
**Cockpit-Llama**	**LLM**	**71.32**	**80.01**	**76.89**	**81.42**

**Table 2:** In-context learning and Freeze vs. Cockpit-Llama (LoRA).

Method	Evaluation			
	Bleu-4	Rouge-1	Rouge-2	Rouge-L
In-Context	39.54	36.24	44.02	42.29
Freeze	46.88	49.45	54.32	50.80
**Cockpit (Lora)**	**71.32**	**80.01**	**76.89**	**81.42**

## Data Availability

The data presented in this study are available upon request from the corresponding author.
